# The Role of Matrix Gla Protein in Ossification and Recovery of the Avian Growth Plate

**DOI:** 10.3389/fendo.2012.00079

**Published:** 2012-07-10

**Authors:** Harel Dan, Stav Simsa-Maziel, Adi Reich, Dalit Sela-Donenfeld, Efrat Monsonego-Ornan

**Affiliations:** ^1^Institute of Biochemistry and Nutrition, The Robert H. Smith Faculty of Agriculture, Food and Environment, The Hebrew UniversityRehovot, Israel; ^2^Koret School of Veterinary Medicine, The Robert H. Smith Faculty of Agriculture, Food and Environment, The Hebrew UniversityRehovot, Israel

**Keywords:** chondrocytes, MGP, BMP2, thiram, tibial dyschondroplasia

## Abstract

Extracellular matrix mineralization is an essential physiologic process in bone, teeth, and hypertrophic cartilage. Matrix Gla protein (MGP), an inhibitor of mineralization, is expressed by chondrocytes and vascular smooth muscle cells to inhibit calcification of those soft tissues. Tibial dyschondroplasia (TD), a skeletal abnormality apparent as a plug of non-vascularized, non-mineralized, white opaque cartilage in the tibial growth plate of avian species can serve as a good model for studying process and genes involved in matrix mineralization and calcification. In this work, we studied the involvement of MGP in the development of TD, as well as in the processes of spontaneous and induced recovery from this syndrome. First, we found that during normal bone development, MGP is expressed in specific time and locations, starting from wide-spread expression in the yet un-ossified diaphysis during embryonic development, to specific expression in hypertrophic chondrocytes adjacent to the chondro-osseous junction and the secondary ossification center just prior to calcification. In addition, we show that MGP is not expressed in the impaired TD lesion, however when the lesion begins to heal, it strongly express MGP prior to its calcification. Moreover, we show that when calcification is inhibited, a gap is formed between the expression zones of MGP and BMP2 and that this gap is closed during the healing process. To conclude, we suggest that MGP, directly or through interaction with BMP2, plays a role as ossification regulator that acts prior to ossification, rather then simple inhibitor.

## Introduction

Longitudinal growth takes place at the growth plates containing cartilage cells (chondrocytes) at different stages of differentiation, which are gradually replaced by bone tissue through endochondral ossification process (Hunziker, 1994). Chondrocytes within the growth plate can be divided into different anatomic zones according to their morphology, components of the extracellular matrix (ECM), enzymes activity, and expression of growth factors and receptors. The resting zone, in which the ratio of ECM to cell volume is high and the cells are in a relatively quiescent state; the proliferative zone, in which the chondrocytes have a flattened-spheroid shape and arranged into columns, forming dense structures parallel to the bone’s longitudinal axis, and produce matrix rich in type II collagen (Col II); the pre-hypertrophic zone in which chondrocytes cease to proliferate and begin to undergo differentiation; the hypertrophic zone containing fully differentiated chondrocytes with large cellular volume, matrix rich in type X collagen (Col X), and alkaline phosphatase (ALP) activity; the ossification zone, which is invaded by blood vessels, the ECM undergo mineralization, and replaced by bone tissue (Olsen et al., 2000; Kronenberg, 2003).

Extracellular matrix mineralization is an essential physiologic process in bone, teeth, and hypertrophic cartilage. To date, few proteins acting as regulators of ECM mineralization have been identified; all are considered as mineralization inhibitors. These include: Ank, a protein controlling export of pyrophosphate (a small molecule that itself inhibits ECM mineralization); NPPS, an ectoenzyme generating pyrophosphate; and matrix gla protein (MGP) (Jahnen-Dechent et al., 1997; Okawa et al., 1998; Nakamura et al., 1999; Ho et al., 2000; Schafer, 2003).

Matrix Gla protein is expressed in chondrocytes and in vascular smooth muscle cells, two cell types that produce an un-calcified ECM (Luo et al., 1997). It contains gla (γ-carboxylated glutamic acid) residues (Pudota et al., 2000; Bandyopadhyay et al., 2002), and a posttranslational modification confers the protein high affinity for hydroxyapatite crystals which are the major mineral crystal present in mineralized ECMs (Romberg et al., 1986; Roy and Nishimoto, 2002). This enables MGP to bind mineral crystals, thereby actively inhibiting calcification of the soft tissues in which it is expressed. MGP-deficient mice develop spontaneous calcification of arteries and cartilage, and exhibit inappropriate calcification of the growth plate, which eventually leads to short stature, osteopenia, and fractures (Luo et al., 1997).

The avian growth plate, due to its unique feature, can serve as a model to study growth-plate mineralization and ossification. In this plate the chondrocytes columns are higher, denser, and less oriented than in mammals, hence it is longer and more vascularized than the mammalian one (Leach and Gay, 1987; Hunziker, 1994). Moreover, disruptions in calcification of the growth plate can lead to skeletal abnormalities such as tibial dyschondroplasia (TD) and rickets, two disorders causing bone fractures and deformities, and can serve as an exceptional model to study calcification-related issues. Aside for the model benefits that enable to study the role of various genes and proteins in the calcification process, these species differences may influence the results, serving on one hand as general developmental model while also may reflect avian specific phenomenon.

Tibial dyschondroplasia is one of the most prevalent skeletal abnormalities observed in avian species that have been genetically selected for rapid growth. It was first described by Leach and Nesheim (1965) as an apparent plug of non-vascularized, non-mineralized, white opaque cartilage in the tibial growth plate. Although the cause of TD is still not known, several theories were suggested to explain its’ etiology including: inhibition in the differentiation of pre-hypertrophic chondrocytes to hypertrophic (Leach and Gay, 1987; Farquharson et al., 1992; Farquharson and Jefferies, 2000; Praul et al., 2000; Webster et al., 2003), secretion of immature cartilage resistant to resorption and vascularization (Orth and Cook, 1994); defects in the synthesis and secretion of MMPs resulting in lesser ECM degradation and fewer blood vessels penetration into the growth plate (Simsa et al., 2007b; Hasky-Negev et al., 2008; Dan et al., 2009), and endoplasmic reticulum stress in growth plate chondrocytes that activates autophagy as a cell survival mechanism (Leach and Monsonego-Ornan, 2007). Spontaneous recovery from TD was recorded in poultry; however no attempt was made to understand the mechanism underlying this healing process.

In this work we used a model for induction and recovery from TD. We found that MGP is expressed in the TD lesion during the healing process, and that it is expressed in close proximity to BMP2 in the healing lesion but not in the control growth plate. The results obtained in this study suggest that MGP is not only an inhibitor but rather a regulator of cartilage calcification in the growth plate.

## Materials and Methods

### Animals and induction of TD and rickets

**Figure 1:**
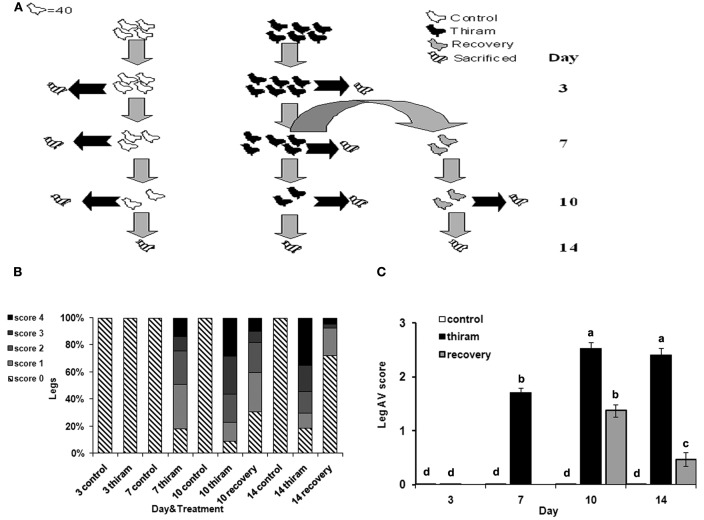
**Effect of thiram and thiram-removal on TD incidence in broilers**. **(A)** Four hundred chicks were divided into two groups: control group received standard diet (*n* = 160) and thiram group which received the same diet containing 50 mg/kg of thiram (*n* = 240). After 7 days, 80 birds from the thiram group were picked randomly and transferred to a non-thiram diet for further 7 days (recovery). At days 3, 7, 10, and 14, 40 chicks from each group were scarified. All tibial growth plates were dissected and scored for TD severity. **(B)** Relative percentages of each score. **(C)** Average score for each group.

One-day-old Cobb strain broiler chicks (*n* = 400) were obtained from a commercial hatchery (Brown Hatcheries, Hod Hasharon, Israel). Chicks were divided into two groups: control group received standard diet according to National Research Council (NRC) recommendations (*n* = 160) and “thiram” group which received the same diet plus 50 mg/kg of thiram (*n* = 240). After 7 days, 80 birds from the thiram group were randomly picked and transferred to a non-thiram diet (Figure 1). All groups were raised for total 14 days under the recommended temperature regime; all groups had free access to food and water. Rickets was induced in 1-day-old Cobb strain broiler chicks (*n* = 50) by supplying a diet with no vitamin D and housing in dark conditions for 15 days. All procedures were approved by the Animal Care Welfare Committee of the ARO’s Volcani Center.

### Validation of TD induction and recovery

On days 3, 7, 10, and 14, the number of lame birds was counted in each group. Forty birds from each group were sacrificed by cervical dislocation; proximal growth plates of the right and left tibia were shaved longitudinally to determine the incidence and severity of TD. The severity was scored subjectively as: 0, healthy growth plate; 1, recognizable cartilage plaque; 2, plaque covering up to 20% of the longitudinal section; 3, plaque covering up to 50% of the longitudinal section; 4, plaque covering up to 80% of the longitudinal section. Whole growth plates were collected and fixed in 4% PFA, or frozen for further analysis.

### RNA isolation and reverse transcription

For RNA extraction, proximal tibial growth plates were isolated and submerged in RNeasy Maxi kit (Qiagen, Hilden, Germany) according to the manufacturer’s protocol. Total RNA (1 μg) was reverse-transcribed in a final volume of 20 μl with the Reverse-RT kit (ABgene, Epsom, Surrey, UK) using oligo-dT/hexamer primers, at reaction temperatures of 42°C for 1 h and 75°C for 10 min.

### Real-time PCR

cDNA (1 μl) was used for real-time PCR using the fluorescent dye SYBR Green I (Absolute QPCR SYBR Green Mix, ABgene) to monitor DNA synthesis using specific primers for MGP and BMP2:

MGP – forward: CAGGAGAGGATCAGGGAACG backward: AAGCAGCAGGATAGCCATGG

BMP2 – forward: ACAGTTGCAAAAGGCATCCG backward: CGGAAAAGGACATTCCCCAT

The PCR was carried out in the ABI Prism 7300 system (Applied Biosystems, Foster City, CA, USA) using the following cycling protocol: a 95°C denaturation step for 15 min followed by 40 cycles of 95°C denaturation (15 s), 60°C annealing (30 s), and 72°C extension (30 s). Gene expression was normalized to the housekeeping gene *Gallus gallus* ribosomal 18S. The amplified PCR product was analyzed with ABI Prism 7300 software (Applied Biosystems). At the end of the real-time PCR, a melting curve was determined to verify the presence of a single amplicon (Reich et al., 2008).

### Preparation of probes

Probes for *in situ* hybridization were prepared by PCR amplification from cDNA of both chicken growth plates and primary cultured chondrocytes isolated from the proximal tibial growth plates using the following primers:

MGP – forward: AATGCGTGCTCTCATCGTC backward: TCCTCCTCCCAAAATAGTGC

BMP2 – forward: AAGTGGGAAAACAGCACGAC backward: GATATGGTTGTGGAGGGCTG

Probe sizes ranged from 600 to 800 bp. Products were ligated into pGEM constructs using the pGEM T Easy kit (Promega, Madison, WI, USA) according to the manufacturer’s protocol, to be used as probes for *in situ* hybridization. Restriction enzymes were used to create sense or antisense probes (Tong et al., 2003; Simsa et al., 2007a).

### Histological staining and *in situ* hybridization of growth-plate sections

Growth plates were fixed overnight in 4% paraformaldehyde (Sigma) in PBS at 4°C. The samples were dehydrated in graded ethanol solutions, cleared in chloroform, embedded in Paraplast, and 5-μm sections were prepared. Alcian-blue and Von-Kossa (Ab&Vk) staining were performed with 0.6% Alcian-blue 8 GX in 70% ethanol and with 2% silver nitrate exposed to sunlight. Hybridizations were performed as described by Reich et al. (2005). The sections were deparaffinized in xylene, rehydrated through a graded series of ethanol solutions, rinsed in distilled water (5 min), and incubated in 2 × SSC at 55°C for 30 min. The sections were then rinsed in distilled water and treated with proteinase K (10 μg/ml in 0.2°M Tris-HCl, 5 mM EDTA, pH 7.5) for 10 min. After digestion, slides were rinsed with distilled water, fixed in 10% formaldehyde in PBS, blocked in 0.2% glycine, rinsed in distilled water, rapidly dehydrated through graded ethanol solutions, and air-dried for several hours. The sections were then hybridized with ^35^S-labeled probes (10 ng) for MGP, or BMP2, (Reich et al., 2005). Radioactive signals were intensified using emulsion (Eastman Kodak Company, Rochester, NY, USA): the sections were incubated with the emulsion solution for 1 month, in the dark, at room temperature. No signal was observed in any of the hybridizations with sense probes, which were used as controls.

## Results

### Thiram-induced TD lesion is reversible through a recovery process

To induce TD, chicks were fed with thiram-enriched diet (thiram group) according to well established protocol (Rath et al., 2004; Hasky-Negev et al., 2008). After 1 week, half of the chicks in this group were picked randomly and the thiram-enriched diet was replaced to normal diet for another week in order to obtain a recovery process, while the other half continued to receive thiram (Figure 1A). During the 2-weeks experimental period, growth plates were collected for TD scoring, RNA preparation, and histological analysis.

The results of TD scoring (Figure 1B), demonstrate that after 3 days of thiram consumption no growth plates were affected by TD. However, on the 7th day, 83% of the legs had TD of any severity. After 10 days the percentage of disordered growth plates was 94%, and after 2 weeks in the thiram group it was 81% (due to spontaneous recovery). There were no cases of TD in the control group during all the experiment. The data in Figure 1C presents the average score per group. This, together with the data in Figure 1B, demonstrates the worsening of the lesion with the progression of the experiment. On day 7, the average score for the thiram group was 1.7 ± 0.09 as only 25% of legs had severe TD lesions (scored 3 and 4) while after 10 days the average score was 2.53 ± 0.11 resulting from 60% legs with severe lesions. The recovery group showed lower levels of TD occurrence and severity of lesions, already after 3 days of recovery (day 10), at which the average score was 1.38 ± 0.12, representing 18% of the legs with severe lesions (score 3 and 4) and 50% with milder TD (score 1 and 2). This was further improved after 7 days of recovery (day 14) at which the average score was 0.47 ± 0.13 as no more than 7% of the legs had severe lesions and 20% had milder TD, while the average score of the thiram group at that time was 2.41 ± 0.12. The presented data validates the quality and the stability of our model.

### MGP is expressed in a pre-ossification manner during bone development

In order to find genes which are involved in the development and recovery of TD, mRNAs were extracted from growth plates of each group, and tested for differentially expressed genes between the groups. One of those genes was MGP, a vitamin K-dependent matrix calcification inhibitor (Luo et al., 1997).

**Figure 2:**
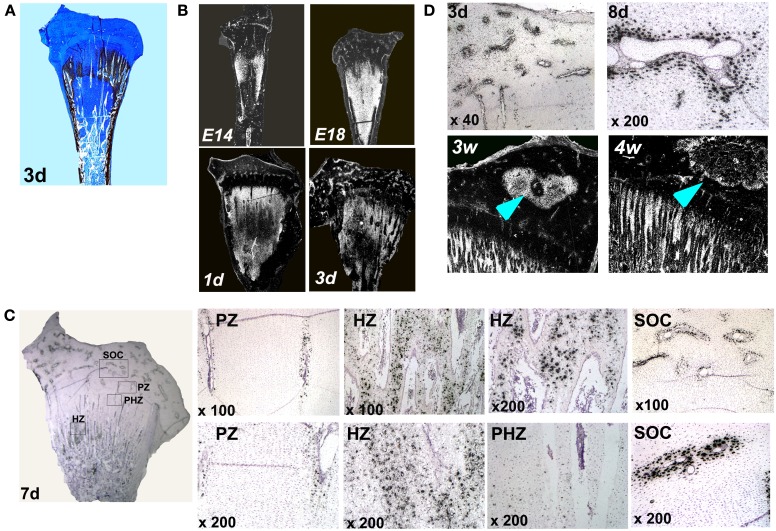
**Matrix Gla protein expression in the chick’s growth plate during development**. **(A)** Alcian-blue & Von-Kossa staining of whole growth plate at the age of 3 days. **(B)** Chick growth plates from embryonic days 14 and 18 and at the ages of 1, 3, and 7 days were processed as described in Section “Materials and Methods” and subjected to *in situ* hybridization with 35S-labeled riboprobes of the avian MGP (×7.5). **(C)** Expression of MGP in the growth plate of 7 days old chick. PZ, proliferative zone – MGP expression around blood vessels in the proliferative zone; HZ, hypertrophic zone – expression in hypertrophic chondrocytes; PHZ, pre-hypertrophic zone – no expression in the pre-hypertrophic zone; SOC, secondary ossification center – MGP expression around epiphyseal blood vessels. **(D)** MGP expression during the formation of the secondary ossification center. On days 3 and 8 (3d and 8d) MGP is expressed prior to ossification in cells surrounding the blood vessels of the epiphysis. On 3 and 4 weeks (3w and 4w), once ossification takes place, MGP expression in the margins of the secondary ossification center (arrows). (×10).

**Figure 3:**
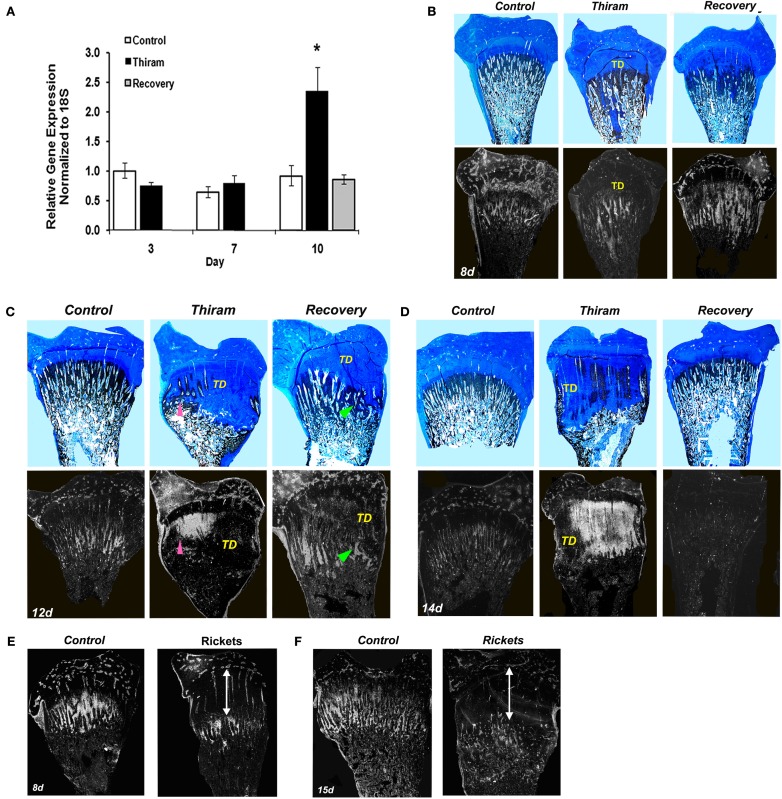
**Matrix Gla protein expression in the processes of TD development and recovery**. **(A)** RNA was extracted from growth plates of the control, thiram, or recovery groups on days 3, 7, and 10. MGP expression was measured by real-time PCR analysis. **(B–D)** Whole growth plates from control, thiram, or recovery groups on days 8 **(B)**, 12 **(C)**, and 14 **(D)** were stained with Alcian-blue & Von-kossa (upper panels) or processed as described in Section “Materials and Methods” and subjected to *in situ* hybridization with ^35^S-labeled riboprobes of the avian MGP (lower panels; ×7.5). **(B)** No MGP expression in the lesion area. **(C,D)** Arrows pointing MGP expression in areas that show signs of healing and resorption. TD, tibial dyschondroplasia. **(E,F)** Rickets was induced by vitamin D deficiency. MGP expression was studied on days 8 **(E)** and 15 **(F)** by *in situ* hybridization. No MGP expression was found in the rachitic lesion (marked by an arrow), its expression is “pushed” to the new chondro-osseous junction.

Up until now, localization of MGP transcripts in the tibial growth plate was described only in a 12-days-old chicken embryo (e12) (Yagami et al., 1999), however detailed characterization of MGP expression during the development of the chicken’s growth plate has not been published. For this purpose, we applied an *in situ* hybridization analysis on the proximal tibia of e14, e18, 1, 3, 7, 8, 12, and 14 days old chickens. Ab&Vk staining, in which cartilaginous substance stains blue and minerals stains black, were performed in order to localize MGP expression (Figures 2 and 3). Up to the age of 3 days after hatching there are still vast un-ossified cartilaginous regions in the developing bone (Figure 2A). In those stages MGP is expressed throughout the pre-ossified cartilage and around blood vessels of the epiphysis (Figure 2B). From the 7th day the metaphysis is fully ossified and a defined growth plate had already formed (Dan et al., 2009). Detailed investigation of MGP’s expression on this day reveals expression in cells surrounding the blood vessels of the proliferative zone but not in proliferative chondrocytes (Figure 2C – PZ), while in the hypertrophic zone the opposite phenomena is observed – hypertrophic chondrocytes strongly express MGP, except for those adjacent the blood vessels (Figure 2C – HZ). The expression of MGP is particularly observed near the chondro-osseous junction. No MGP expression was seen in the pre-hypertrophic zone (Figure 2C – PHZ). In the secondary ossification center (SOC), MGP’s expression pattern was similar to that seen in the proliferative zone, meaning expression in cells located in close proximity to the blood vessels (Figure 2C – SOC). This pattern characterizes MGP expression in all further developmental stages we checked (Figures 3B–D). These results suggest that during normal bone development MGP is expressed in areas that are about to undergo ossification immediately prior to its initiation.

The expression pattern of MGP, preceding the ossification process is demonstrated very clearly during the formation of the SOC. The SOC is formed by endochondral ossification, i.e., its development begins by the formation of a cartilage template which is later replaced by bone (Rivas and Shapiro, 2002). In early stages of the SOC development (days 3 and 8), prior to its ossification, when it is still occupied by cartilage, MGP is expressed throughout the epiphysis in the place where ossification later occurs (Figure 2D). Once ossification takes place (3 and 4 weeks), MGP expression diminishes from the center and accompanies the margins of the SOC (Figure 2D).

### MGP expression is increased within the healing TD lesion and precedes the ossification process

Next we were interested to understand whether MGP is involved in the development of TD and the process of recovery from it. For that purpose we quantified MGP’s expression in growth plates isolated from the three experimental groups by real-time PCR. As expected, on day 3, when the lesion is only initiating, MGP expression was not different between the thiram and the control groups (Figure 3A). However, on day 10, when 60% of the legs in the thiram group have severe TD lesions (Figure 1B), MGP expression is almost 2.5 times higher in the thiram-fed chicks compared to the control (Figure 3A), suggesting that MGP is involved in the inhibited calcification of the TD lesion. Interestingly, in the recovery group, MGP expression level is not different from the control (Figure 3A), meaning that no more than 3 days after the removal of thiram, MGP expression returns to normal, allowing ossification, and thereby recovery of the lesion.

Since prior to this study MGP had never been associated with TD, we further established its involvement in the development and recovery from this syndrome, by studying MGP’s expression in sequential sections stained with Ab&Vk applied on a set of longitudinal sections of proximal tibias isolated from the different groups (Figures 3B–D). On the 8th day (Figure 3B), most of the metaphysis is ossified, both in the control and thiram groups. Correspondingly, a drastic reduction in MGP expression is observed compared to day 3 (Figure 2B). This reduction is expected since MGP’s role is to inhibit calcification in non-calcified tissues (i.e., cartilage), once the tissue had gone calcification, MGP is no longer needed. On this day, MGP expression is found in the proximity of blood vessels in the epiphysis and proliferative zone, as well as in the hypertrophic chondrocytes mainly those that are close to the chondro-osseous junction, as shown in Figure 2C. In the thiram group, the TD lesion is already apparent on this day. MGP expression is not seen in the lesion area, but it appears below the lesion, where calcification takes place (Figure 3B). The recovery process is only initiating (24 h), with strong MGP expression in the place soon to be ossified (Figure 3B). On the 12th day the lesion is wide-spread over the metaphysis containing dense opaque cartilage as observed by Alcian-blue staining. Spontaneous recovery process begins at the margin of the lesion characterized by partial resorbtion and ossification seen as limited Von-Kossa staining surrounded by cartilaginous material (Figure 3C pointed with arrowhead). MGP is not expressed in the dense lesion but it is highly expressed in the area undergoing recovery (Figure 3C lower panel pointed with arrowhead). In the recovery group, at this time point, the recovery process is in its peak (Figure 3C); a lesion is still observed, however it is much smaller than the lesion in the thiram group. The Von-Kossa staining, which replaced the Alcian-blue staining, together with the evident blood vessels penetrating the growth plate (Figure 3C), point that the lesion is undergoing rapid resorption and ossification. Similar to the observed during the spontaneous recovery, here we also see that MGP is absent from the opaque lesion area but it reappears at the healing site (Figure 3C). On day 14, an interesting phenomena is observed, MGP which up till now was absent from the lesion, is widely expressed within the lesion of the thiram group (Figure 3D). On this day, massive spontaneous recovery occurs in the thiram group, revealed by the ossifying cartilage (Von-Kossa staining) inside the lesion (Figure 3D), and blood vessels penetration into it. Note that MGP is particularly expressed in areas that show signs of healing and resorption (Figure 3D pointed with arrowhead). In the recovery group, the lesion is completely ossified by this day, and resembles the control, and so is MGP expression which is hardly detectable in this group (Figure 3D).

For comparisons, we studied the expression of MGP in rachitic growth plates as another model of calcification impedance, but unlike the TD lesion, it does not go a recovery process. Rickets was induced by vitamin D deficiency and housing in dark conditions (Hasky-Negev et al., 2008). The rachitic growth plates at the ages of 8 (Figure 3E) and 15 days (Figure 3F) demonstrated the typical phenotype of expanded growth plate due to inhibition of mineralization. MGP was expressed along the chondro-osseous junction, but not within the rachitic lesion (Figures 3E,F), demonstrating that MGP is expressed in remodeled cartilaginous lesions but not in persistence cartilage lesion that does not resolved. This strengthens our suggestion that MGP plays a significant role in regulating the calcification process in a wider sense than inhibition through calcium binding.

**Figure 4:**
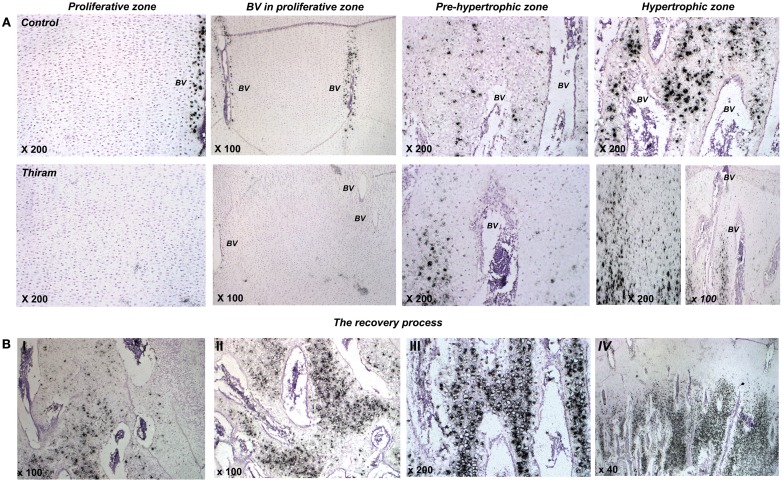
**Thiram effects cell morphology and MGP expression in the growth plate**. **(A)** The effect of thiram on MGP expression in the proliferative zone, around blood vessels in the proliferative zone, pre-hypertrophic, and hypertrophic zones. **(B)** Stages of the recovery process. I, most impaired; IV, most healed.

Higher magnifications demonstrate thiram’s effect on cell morphology and MGP expression (Figure 4A). In the proliferative zone cell morphology appears normal (Figure 4A), however MGP expression in the cells surrounding the vessels is lower in the thiram group (Figure 4A – BV in proliferative zone). In the pre-hypertrophic (Figure 4A) and hypertrophic zones (Figure 4A), cell morphology and spatial organization is dramatically changed following thiram treatment; the normal well organized structure of cells become disoriented and scattered with abnormal cell morphology and scattered MGP expression (Figure 4A). Moreover, as expected, the TD lesion is clear of blood vessels (Figure 4A lower panel – left image). Vessels do not penetrate into the lesion but rather stop at its border creating a truncated shaped vessel (Hasky-Negev et al., 2008). Figure 4A lower panel – right image demonstrates an impaired area with no blood vessels and no MGP expression, and next to it, a healed area in which blood had already penetrated to and MGP is expressed (Figure 4A lower panel – right image).

During the recovery process (whether spontaneous or deliberate) MGP is expressed in areas that are about to be calcified (Figures 3C,D). The recovery is not a homogenous process throughout the lesion; different stages of healing are noticed in the recovered growth plate. Figure 4B shows changes in MGP expression demonstrating those stages. The stages are marked I, the most impaired, to IV, the most healed. Figure 4BI demonstrates an area where the lesion is still wide-spread based on the cells abnormal morphology and the few blood vessels which are truncated at the lesion border. No MGP expression is found in the impaired area. On the left side of the image scattered MGP expression start to appear. Figure 4BII was taken from an area at the lesion border. More blood vessels are observed, but those are still not organized, and more cells express MGP. The area in Figure 4BIII is almost healed; normal-appearing blood vessels are found in this area and strong MGP expression is observed in hypertrophic cells before calcification. Figure 4BIV represents a fully healed growth plate; metaphyseal blood vessels penetrate up to the pre-hypertrophic zone and MGP expression is strong in the hypertrophic zone in a similar way to the control growth plates.

Our results demonstrate that MGP is not expressed randomly by various cells in non-calcified tissues; on the contrary it has a specific timing and pattern of expression. Based on these results we suggest that MGP is not merely a calcification inhibitor, it is expressed prior to ossification and might regulate the initiation of the calcification process.

### Proximity between MGP and BMP2 expression zones in the growth plate during healing

**Figure 5:**
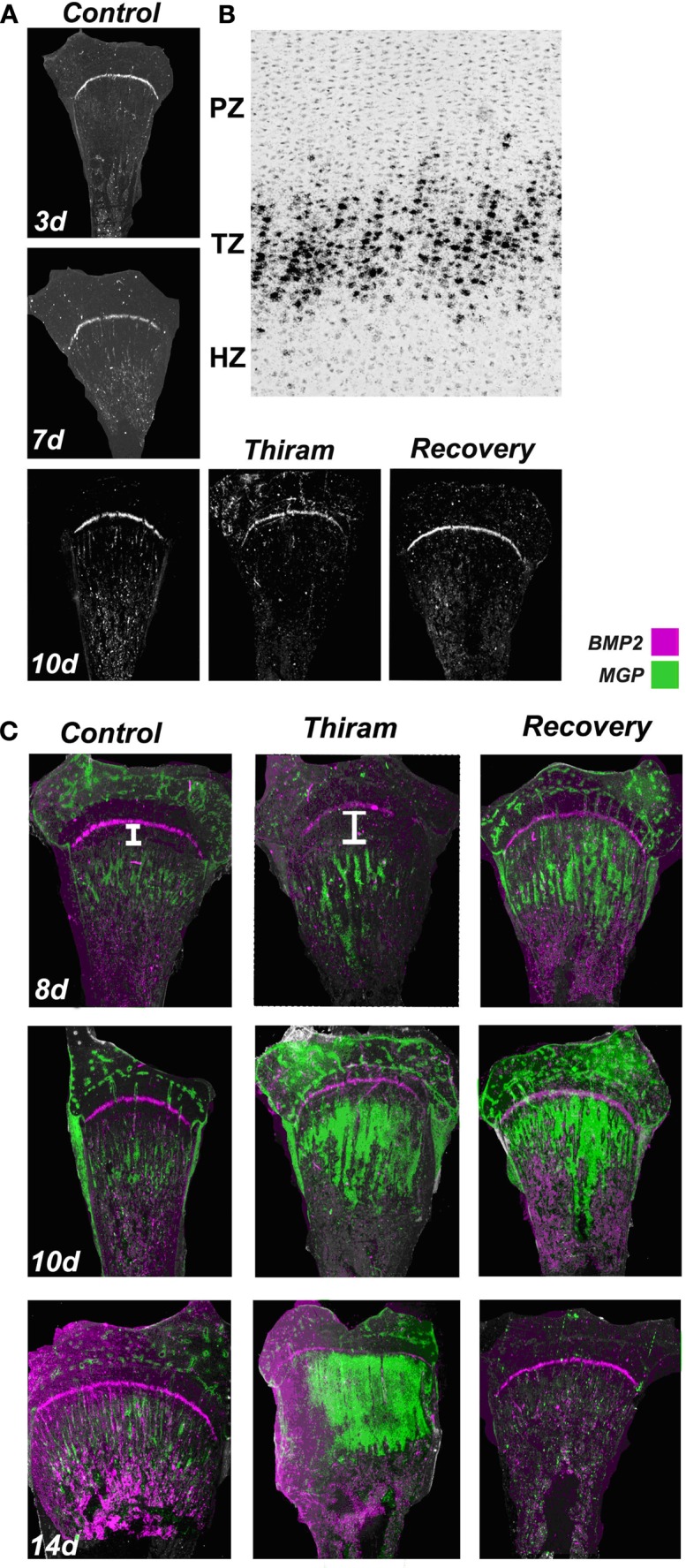
**Proximity between the expression zones of BMP2 and MGP in the growth plate**. Whole growth plates were processed as described in Section “Materials and Methods” and subjected to *in situ* hybridization with ^35^S-labeled riboprobes of the avian BMP2 or MGP. **(A)** BMP2 expression on days 3, 7, and 10 is restricted to the pre-hypertrophic (transition zone). BMP2 expression is not influenced by thiram or recovery (×7.5). **(B)** BMP2 expression in the transition zone (TZ). Bright field (×100). **(C)** Overlap of MGP (green) and BMP2 (purple) expression in control, thiram, and recovery (×7.5). White line in the upper panel demonstrates the gap formed between the expression zones of these two genes in the thiram group compared with the control.

Back in the 1980s, when BMP was first discovered by Kawamura and Urist (1988), they reported it had a tight association with MGP, and suggested that MGP could influence the delivery of BMP to target cells through formation of a MGP-Ca^2+^-BMP aggregate, and more recent studies have supported this hypothesis (Bostrom et al., 2001; Sweatt et al., 2003). In order to check whether such interaction is probable in the avian growth plate, we studied BMP2 expression in different developmental stages of the growth plate as well as in the TD lesion and during recovery from TD (Figure 5A). Hybridizations from days 3, 7, and 10 show BMP2 expression does not change during development, and is exclusively expressed by pre-hypertrophic chondrocytes (transition zone, TZ) in all groups (Figures 5A,B). Moreover, BMP2 expression is not influenced by thiram or thiram-removal (Figure 5A – 10d).

To further explore possible interaction and regulation of BMP2 by MGP, we studied the co-localization of MGP (green) and BMP2 (purple) transcripts in the growth plates (Figure 5C). On day 8, the control growth plates demonstrated a thin line of BMP2 expression in the pre-hypertrophic zone, and MGP expression in the pre-ossified chondro-osseous junction. In the thiram group, MGP expression was pushed to the lesion boundaries opening a large gap between BMP2 and MGP expression zones. Interestingly, in the recovery group, with the initiation of the recovery process, this gap was closed and the expression zones of BMP2 and MGP were in close proximity again (Figure 5C – 8d). Same pattern was observed on day 10 in the recovered growth plates, with reduced gap between MGP and BMP2 expression (Figure 5C – 10d). On day 14, BMP2 expression is not changed and MGP expression is almost vanished from the control group. In the thiram group very interesting phenomena is observed, the cells in the lesion, which go spontaneous recovery, vastly express MGP, closing completely the gap between the expression zones of BMP2 and MGP which almost overlap. In the recovery group, in which by now the lesion had recovered completely, MGP expression is hardly detected (Figure 5C – 14d).

These results, although descriptive, suggest that close proximity between MGP and BMP2 is essential for normal ossification. When a gap is formed between BMP2 and MGP expression zones the ossification process is slow down, and vice versa – during the recovery process, when the gap is reduced ossification is accelerated. This reduced gap brings BMP2 and MGP to close proximity and enable an interaction between their gene products. These results raise the speculation that MGP has a dual role in the growth plate, both, as calcification regulator and, as a modulator of BMP.

## Discussion

Ossification inhibition might be one of the mechanisms leading to the development of the characteristic TD plaque. Up till now a few candidates have been described as inhibitors of matrix mineralization and bone formation (Leach and Monsonego-Ornan, 2007). In this paper we show, for the first time, a link between one of those inhibitors – MGP and TD. Moreover, we studied the localization of MGP mRNA during the development of the avian growth plate and showed that during late stages of embryogenesis, MGP is widely expressed in the pre-ossified cartilage anlagen, but after hatching, its expression gradually diminishes into the lower hypertrophic zone next to the forming bone as well as in cells surrounding the blood vessels of the proliferative zone and the epiphysis. These expression zones are distinguished from those reported for mouse MGP, in which the gene is expressed mainly in the proliferating and to much lower extents in the late hypertrophic zone of the growth plate (Luo et al., 1997). Furthermore, we studied MGP expression during the development of the SOC and found that it is highly expressed in the center of the “future SOC” and than diminished from the center to the surroundings of the ossified zone. These results demonstrate that in the avian growth plate, MGP is expressed specifically in a pre-ossification manner and suggest that it acts as an ossification regulator, rather than as a simple ossification inhibitor.

To further address this issue we studied MGP expression in a model for impaired ossification – the thiram-induced TD lesion and the process of recovery from TD induced by thiram-removal. We found that in early stages of TD development (day 8), MGP is not expressed in the lesion cells and it appears as if “pushed aside” to the new chondro-osseous junction adjacent to the lesion boundaries. In later stages (days 12 and 14), in parallel to the spontaneous recovery process that begins despite continues presence of thiram, MGP is vastly expressed in the healing lesion. Interestingly, the cells which express MGP are those found in areas undergoing healing process, prior to its ossification. MGP seems to play a significant role in the induced recovery process as well; A day after the removal of thiram (day 8), when the recovery process is only initiated, MGP is strongly expressed in the place soon to be ossified. As the recovery process continues, the lesions are gradually ossified, and MGP expression is found only in the healing sites but absent from the lesions area. With the completion of the recovery, MGP expression is gradually reduced. The consistency between the onsets of recovery (both spontaneous and induced), the morphology of the lesion (partially vascularized or completely opaque), and the specific and timed pattern of MGP expression is our strongest hint to suggest this new function for MGP as calcification regulator.

The role of MGP as calcification inhibitor was established by demonstrating that mice lacking MGP developed spontaneous calcification of arteries and cartilage (Luo et al., 1997). The fact that calcification occurs at these tissues of all, despite the fact that MGP is widely expressed in other tissues as well (Wiedemann et al., 1998), implying that the effect of MGP is developmentally regulated (Yagami et al., 1999). One might argue that the expression of MGP in the TD lesion acts only as a calcification inhibitor. However, the fact that it was not expressed in early stages of the lesion’s development, before it recovers, and in the rachitic lesion which is not healed, but was strongly expressed prior to a recovery process, both spontaneous recovery in late stages in the thiram group and induced recovery after thiram-removal, reveal that this gene does not mark the lesion specifically and suggest that it is indeed ossification regulator rather than merely calcification inhibitor.

The arising complementary issue is how it comes that the lesion does not undergo calcification? If not MGP, what does hinder its ossification? We can speculate that the reason is absence of calcification-inducing agents similar to the case of articular cartilage. There are many resemblances between the articular and the lesion cartilages, in both the prevention of differentiation occurs due to exogenous signals, since isolated chondrocytes from both tissues were able to differentiate in culture (Wu et al., 2005). The lack of differentiation of articular chondrocytes was explained by the lack of differentiation factors due to poor blood vessel penetration into this cartilage. It is possible that a similar mechanism is responsible for the defective differentiation in the lesion (Loveridge et al., 1993; Farquharson et al., 1995). Such differentiation factors could be the retinoic acid (RA) known to affect cartilage development. The fact that RA stimulates not only differentiation but also the expression and activity of MMPs in chondrocytes (Wu et al., 1997; Tong et al., 2003; Simsa et al., 2007b) further support this option.

Previously, we suggested that the TD lesion can be viewed as a survival mechanism in response to stress that targets chondrocytes in the vicinity of the distal proliferative and TZs (Leach and Monsonego-Ornan, 2007). Due to close similarity between the pathological changes associated with TD and those reported by Tsang et al. (2007) for ER stress and autophagy. The avascular TZ in the growth plate, where BMP2 is expressed, appears to be the critical area for the development of these changes. In this paper we characterized, for the first time, the expression pattern of BMP2 in the avian growth plate. In resemblance to its expression in mammalian growth plates, BMP2 was localized to the pre-hypertrophic zone (Yoon and Lyons, 2004), a TZ between two vasculature systems of the growth plate (the epiphyses and the metaphyses; Hunt et al., 1979; Howlett et al., 1984). Based on our results we suggest an optional mechanism by which MGP directly interacts with BMP2 to generate an inactive complex (Bostrom et al., 2001; Sweatt et al., 2003). Our findings suggest that regulation of the physical proximity between MGP and BMP2 expression zones might play a role in calcification inhibition or initiation. We suggest that when a gap is formed between BMP2 and MGP expression zones, calcification is inhibited. This formed gap prevents the binding of MGP to BMP2, allowing BMP2’s stimulating effect on chondrocytes proliferation that could attribute to the impaired ossification of the TD lesion. On the other hand, during the recovery process the gap is reduced, bringing MGP and BMP2 to close proximity. The proximity between these genes enhances the probability for interactions between their products, so that the binding of MGP to BMP2 inhibits the later effect resulting in accelerated ossification. It is important to notice that our results demonstrate the expression patterns of genes rather than proteins which are the active entities. Thus MGP protein and not the mRNA is the one that regulates ossification or binds BMP. The rationale to use the mRNA levels rise from the fact that the cartilaginous growth plate, is packed in its own matrix which trap the proteins expressed and secreted from chondrocytes. Thus we can assume that protein localization is parallel to the gene expression pattern.

To conclude, we show that MGP expression in the avian growth plate is timely and spatially regulated. We show that it is expressed specifically before ossification initiation in healthy and impaired growth plates and suggest that MGP, directly or through interaction with BMP2, plays a role as ossification regulator that acts prior to ossification.

## Conflict of Interest Statement

The authors declare that the research was conducted in the absence of any commercial or financial relationships that could be construed as a potential conflict of interest.
